# Supplementary material for: Study on Structural Evolution, Thermochemistry and Electron Affinity of Neutral, Mono- and Di-Anionic Zirconium-Doped Silicon Clusters ZrSin0/-/2- (n = 6–16)
Source: Int J Mol Sci. 2019 Jun 15;20(12):2933. doi: 10.3390/ijms20122933 (PMC6627843; doi:10.3390/ijms20122933)
Supplement: Supplementary file 1 [file ijms-20-02933-s001.pdf]

Supplementary Information

Study on Structural Evolution, Thermochemistry and Electron Affinity of Neutral, Mono- and Di-Anionic Zirconium-Doped Silicon Clusters $ZrSi_n^{0/-/2-}$ (n = 6–16)

Caixia Dong [1,2], Limin Han [1], Jucai Yang [1,3,*] and Lin Cheng [1,*]

[1] Inner Mongolia Key Laboratory of Theoretical and Computational Chemistry Simulation, School of Chemical Engineering, Inner Mongolia University of Technology, Hohhot 010051, China; Dongcx201011@163.com (C.D.); hanlimin@imut.edu.cn (L.H.)

[2] School of Mining and Technology, Inner Mongolia University of Technology, Hohhot 010051, China

[3] School of Energy and Power Engineering, Inner Mongolia University of Technology, Hohhot 010051, China

* Correspondence: yangjc@imut.edu.cn (J.Y.); lcheng1983@aliyun.com (L.C.); Tel.: +86-0471-657-6145 (J.Y.)

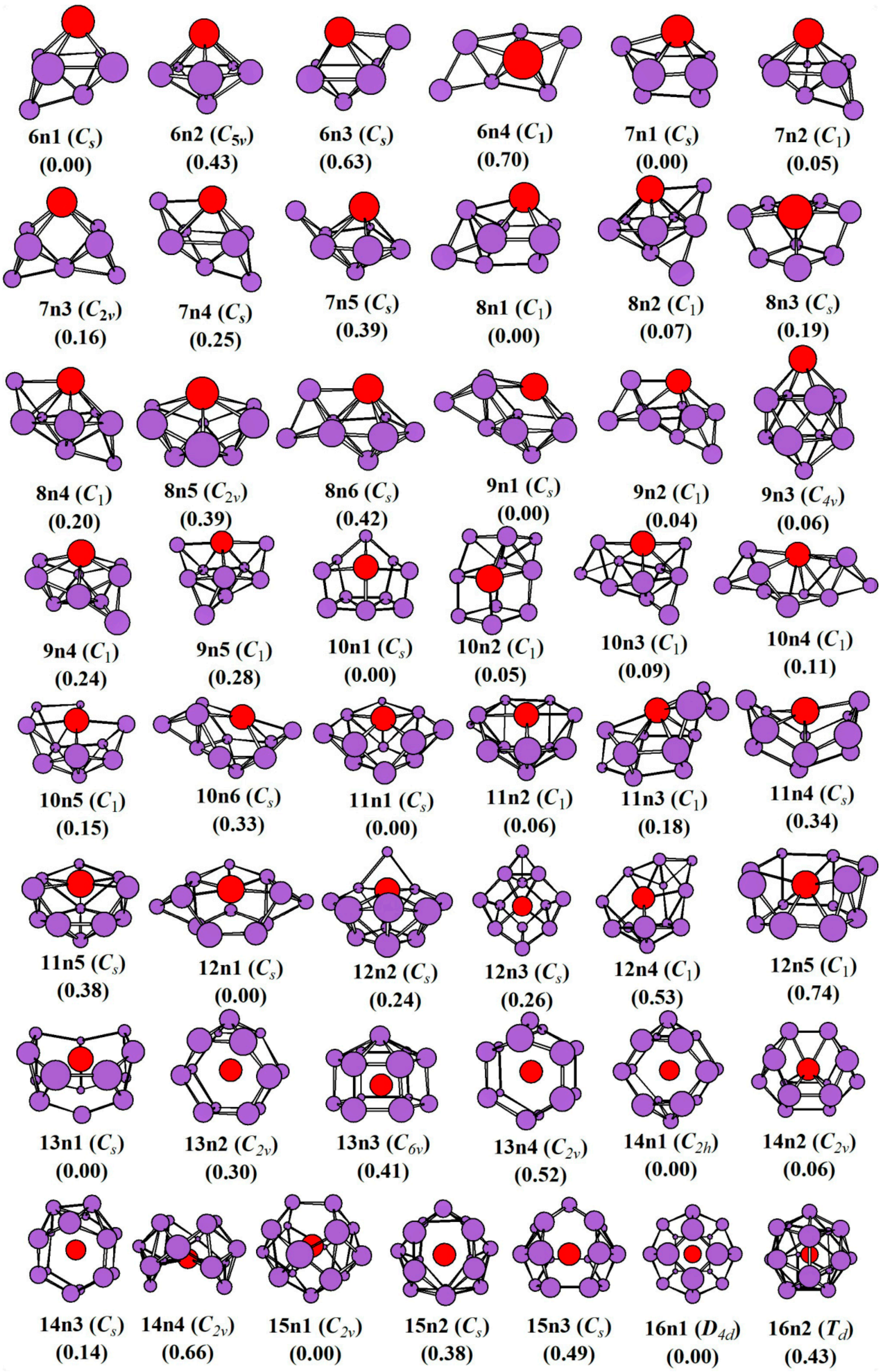

Figure S1. Low-lying isomers of neutral $ZrSi_n$ (n = 6–16) clusters, point group and relative energy (in eV).

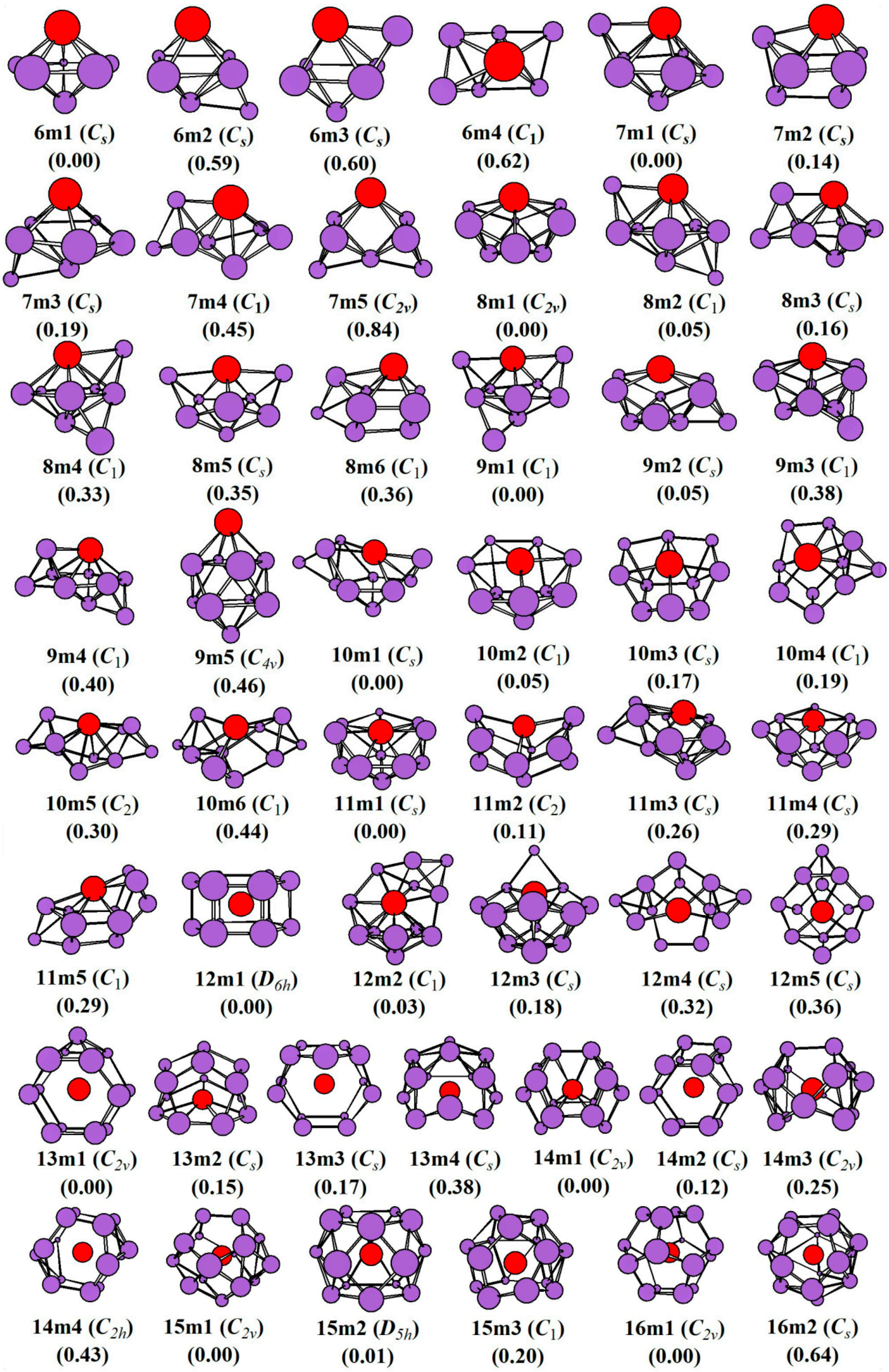

Figure S2. Low-lying isomers of mono-anionic $ZrSi_n^-$ (n = 6–16) clusters, point group and relative energy (in eV).

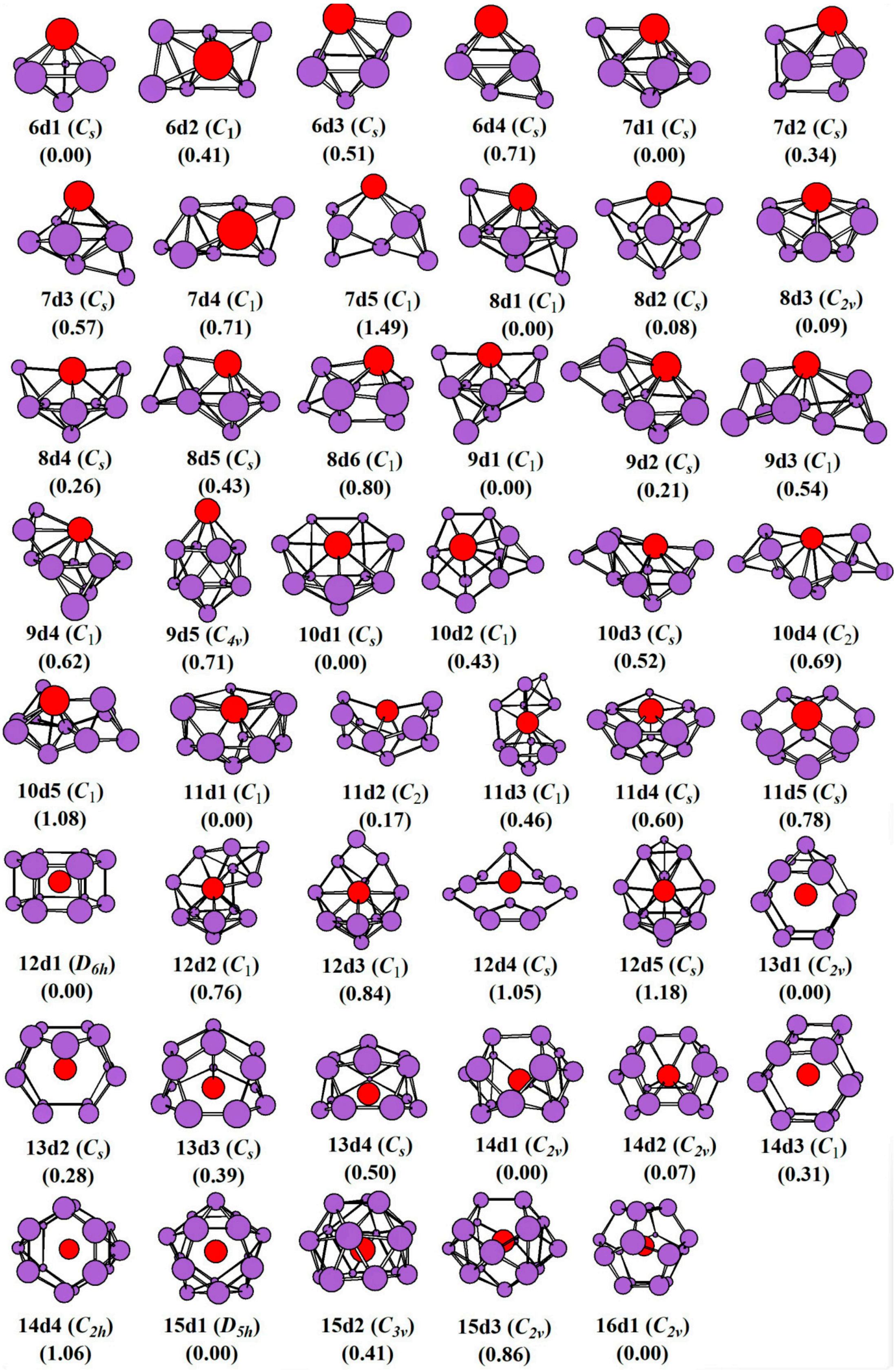

Figure S3. Low-lying isomers of di-anionic $ZrSi_n^{2-}$ (n = 6–16) clusters, point group and relative energy (in eV).

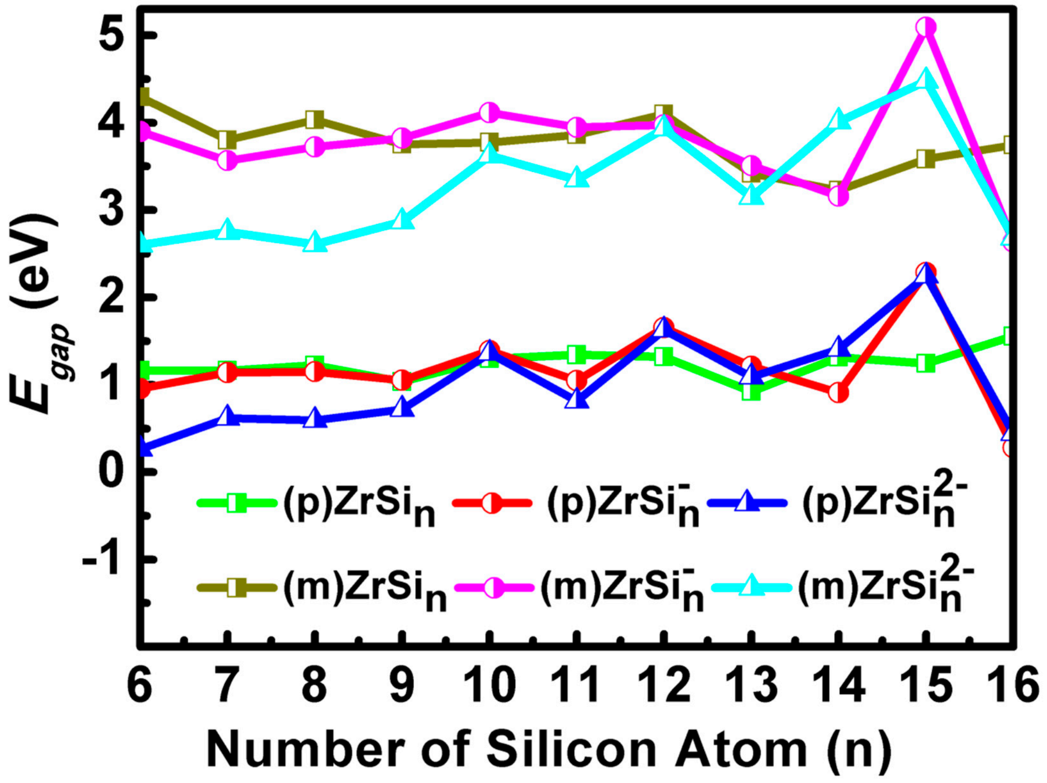

Figure S4. The HOMO-LUMO energy gap (E_{gap}) of $ZrSi_n^{0/-/2-}$ (n = 6–16) clusters. (m) stands for mPW2PLYP calculations and (p) stands for PBEPBE calculations.

Table S1. Conformational population (%) for low-lying geometries of $ZrSi_n^{0/-/2}$ species (n = 6–16) clusters.

Species	Conformational population					
	1	2	3	4	5	6
6n	46.83%	22.65%	16.16%	14.36%		
7n	25.94%	23.84%	19.80%	17.00%	13.42%	
8n	23.06%	20.49%	16.73%	16.45%	11.93%	11.34%
9n	24.22%	22.64%	21.89%	16.15%	15.10%	
10n	20.17%	18.54%	17.33%	16.75%	15.66%	11.55%
11n	26.80%	24.22%	19.78%	15.09%	14.11%	
12n	33.26%	22.18%	21.44%	13.59%	9.53%	
13n	39.71%	23.92%	19.87%	16.50%		
14n	33.10%	29.91%	26.13%	10.86%		
15n	50.93%	26.81%	22.26%			
16n	67.40%	32.60%				
6m	48.00%	17.72%	17.43%	16.85%		
7m	31.01%	24.48%	22.50%	14.50%	7.51%	
8m	22.97%	21.11%	17.53%	13.16%	12.72%	12.51%
9m	29.29%	26.92%	15.42%	14.91%	13.47%	
10m	22.36%	20.55%	16.78%	16.22%	13.47%	10.63%
11m	27.02%	22.44%	17.42%	16.56%	16.56%	
12m	26.21%	24.91%	19.34%	15.27%	14.27%	
13m	32.75%	25.42%	24.58%	17.24%		
14m	33.83%	27.62%	22.18%	16.37%		
15m	37.08%	36.46%	26.45%			
16m	74.67%	25.33%				

6d	44.96%	22.49%	19.00%	13.55%		
7d	42.97%	24.20%	16.41%	12.95%	3.47%	
8d	24.27%	21.20%	20.85%	15.65%	11.74%	6.29%
9d	36.29%	25.45%	14.58%	12.74%	10.94%	
10d	42.15%	20.39%	17.51%	13.14%	6.80%	
11d	35.20%	26.41%	16.18%	12.78%	9.43%	
12d	54.79%	15.18%	13.26%	9.30%	7.47%	
13d	38.90%	24.24%	20.13%	16.72%		
14d	37.77%	33.56%	22.37%	6.30%		
15d	57.66%	28.85%	13.49%			
16d	100.00%					
